# *P. gingivalis* Lipopolysaccharide Stimulates the Upregulated Expression of the Pancreatic Cancer-Related Genes Regenerating Islet-Derived 3 A/G in Mouse Pancreas

**DOI:** 10.3390/ijms21197351

**Published:** 2020-10-05

**Authors:** Daichi Hiraki, Osamu Uehara, Yasuhiro Kuramitsu, Tetsuro Morikawa, Fumiya Harada, Koki Yoshida, Kozo Akino, Itsuo Chiba, Masahiro Asaka, Yoshihiro Abiko

**Affiliations:** 1 Division of Reconstructive Surgery for Oral and Maxillofacial Region, Department of Human Biology and Pathophysiology, School of Dentistry, Health Sciences University of Hokkaido, 1757 Kanazawa, Ishikari-Tobetsu, Hokkaido 061-0293, Japan; daichi0530@hoku-iryo-u.ac.jp; 2 Division of Disease Control and Molecular Epidemiology, Department of Oral Growth and Development, School of Dentistry, Health Sciences University of Hokkaido, 1757 Kanazawa, Ishikari-Tobetsu, Hokkaido 061-0293, Japan; osamu@hoku-iryo-u.ac.jp (O.U.); i-chiba@hoku-iryo-u.ac.jp (I.C.); 3 Research Institute of Cancer Prevention, Health Sciences University of Hokkaido, 1757 Kanazawa, Ishikari-Tobetsu, Hokkaido 061-0293, Japan; climates@hoku-iryo-u.ac.jp (Y.K.); kozo_akino@komei.jp (K.A.); maasaka@hoku-iryo-u.ac.jp (M.A.); 4 Division of Oral Medicine and Pathology, Department of Human Biology and Pathophysiology, School of Dentistry, Health Sciences University of Hokkaido, 1757 Kanazawa, Ishikari-Tobetsu, Hokkaido 061-0293, Japan; t-morikawa@hoku-iryo-u.ac.jp (T.M.); denty@hoku-iryo-u.ac.jp (K.Y.); 5 Division of Oral and Maxillofacial Surgery, Department of Human Biology and Pathophysiology, School of Dentistry, Health Sciences University of Hokkaido, 1757 Kanazawa, Ishikari-Tobetsu, Hokkaido 061-0293, Japan; f-harada93@hoku-iryo-u.ac.jp

**Keywords:** *P. gingivalis*, lipopolysaccharide, pancreatic cancer, *Reg3G*

## Abstract

Although epidemiological studies have shown a relationship between periodontal disease and pancreatic cancer, the molecular mechanisms involved remain unclear. In this study, the effects of systemic administration of *Porphyromonas gingivalis* lipopolysaccharide (PG-LPS) on gene expression were comprehensively explored in mouse pancreas that did not demonstrate any signs of inflammation. PG-LPS was prepared in physiological saline and intraperitoneally administered to male mice at a concentration of 5 mg/kg every 3 days for 1 month. After extracting total RNA from the excised mice pancreas, a comprehensive DNA microarray analysis of gene expression was performed. Tissue specimens were also subjected to hematoxylin–eosin staining and immunohistochemistry using anti-regenerating islet-derived 3A and G (*Reg3A/G*) antibody. ImageJ software was used to quantify the area of *Reg3A/G* positive cells in pancreatic islets by binarizing image date followed by area extraction. The results were compared using Mann–Whitney *U* test. Data are presented as mean ± standard deviation (SD) with *p* < 0.05 considered as significant. *Reg3G*, a gene related to pancreatic cancer, was one of the 10 genes with the highest levels of expression in the pancreas stimulated with PG-LPS. The comprehensive analysis revealed a 73-fold increase in *Reg3G* expression level in the PG-LPS group when compared with the control group; in addition, the expression level of *Reg3A* was increased by 11-fold in the PG-LPS group. Image analysis showed that the ratio of *Reg3A/G* positive cells was higher in the PG-LPS group than the control. Immunostaining showed the presence of *Reg3A/G*-positive cells in the alpha-cell equivalent areas around the islets of Langerhans in the PG-LPS group. These results support the notion that periodontal disease may be a risk factor for pancreatic cancer.

## 1. Introduction

Growing evidence suggests that periodontal disease may be a risk factor for various systemic conditions, such as diabetes, respiratory disease, infectious endocarditis, autoimmune diseases, and chronic kidney disease [1,2,3,4]. Bacteria, inflammatory cytokines, and other inflammation-related components are released into the systemic blood circulation, thus contributing to other systemic diseases. A recent large-scale cohort study found that anti-*Porphyromonas gingivalis* (*P. gingivalis*) antibody titers were higher in pancreatic cancer patients than in healthy subjects, thereby suggesting that periodontal disease might be involved in pancreatic cancer [5]. However, the molecular mechanisms in periodontal disease that may be related to pancreatic cancer have not been identified so far. The 5-year survival rate of pancreatic cancer is one of the lowest among all cancers, and it is the fourth leading cause of cancer deaths in Japan. Chronic pancreatitis and environmental factors such as alcohol consumption and obesity have been identified as potential risk factors for pancreatic cancer [6].

Cerulein has been used to experimentally induce both acute and chronic pancreatitis in mice [7]. In another study, a mouse model of severe acute pancreatitis was developed by inducing with lipopolysaccharides (LPS) derived from *Escherichia coli* (*E. coli*) [8]. These LPS are mainly recognized by toll-like receptor 4 (TLR4), which recognizes Gram-negative bacteria. On the other hand, the Gram-negative periodontal pathogen *P. gingivalis* is recognized by both TLR2 and TLR4 [9,10], which were found to be frequently overexpressed in pancreatic ductal carcinoma [11]. These findings may elucidate the mechanism of action of periodontal disease in the development of pancreatic cancer; however, the manner by which the onset of pancreatic cancer is independently affected by periodontal disease remains unknown. LPS are often used for acute responses in experimental models of acute pancreatitis [8]. Nevertheless, periodontal disease is a chronic inflammatory event and may not cause acute inflammatory changes in the pancreas. We previously developed a mouse model that is unaffected by acute inflammation with *P. gingivalis* lipopolysaccharide (PG-LPS) to observe the effect of LPS on the kidney [12]. The present study aimed to comprehensively explore the effects of systemic administration of PG-LPS on gene expression in the pancreas of these mice.

## 2. Results

### 2.1. Microarray

DNA microarray revealed 1029 probes with more than 2-fold expression and 326 probes with less than 0.5-fold expression following PG-LPS stimulation. The 10 genes with the highest fold change are listed in Table 1. Among them, *Reg3G* has been reported to be related to pancreatic cancer (Table 1) [13].

### 2.2. Gene Expression

**Figure 1:**
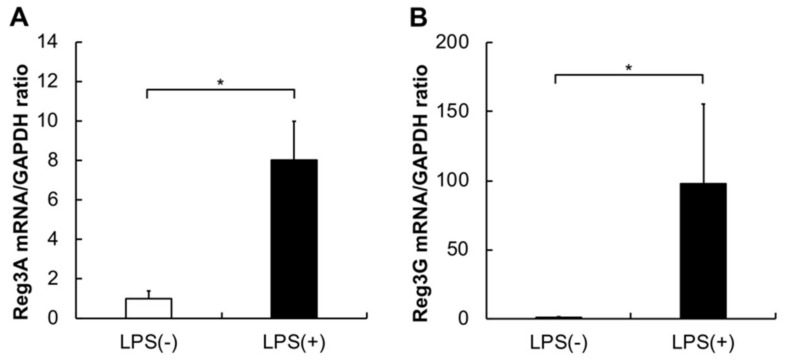
Expression profiles of *Reg3A* and *G* mRNAs in the *P. gingivalis* lipopolysaccharide (PG-LPS) and control mice. *Reg3A* and *G* expression levels were measured by quantitative real-time polymerase chain reaction (qPCR). The expression levels were significantly higher in the PG-LPS mice than in the controls. Data are shown as mean ± standard deviation (SD) obtained from three identical samples, Mann–Whitney *U* test (* *p* ˂ 0.05; *n* = 5). (**A**) *Reg3A* expression levels. (**B**) *Reg3G* expression levels.

The expression levels of *Reg3A* and *G* were significantly higher in the pancreas of mice administered with LPS when compared to those in the controls (Figure 1A,B; *p < 0.05).

### 2.3. Histology, Immunohistochemistry, and Morphological Analysis

**Figure 2:**
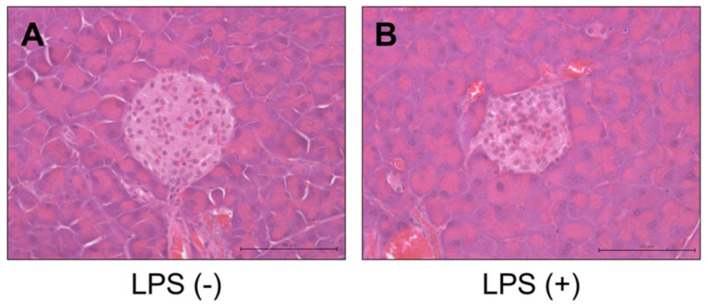
H & E staining of tissues from the *P. gingivalis* lipopolysaccharide (PG-LPS) and control mice. No significant acute inflammatory changes were observed between the control and PG-LPS mice. Scale bar, 100 µm. (**A**) PG-LPS(−). (**B**) PG-LPS(+).

**Figure 3:**
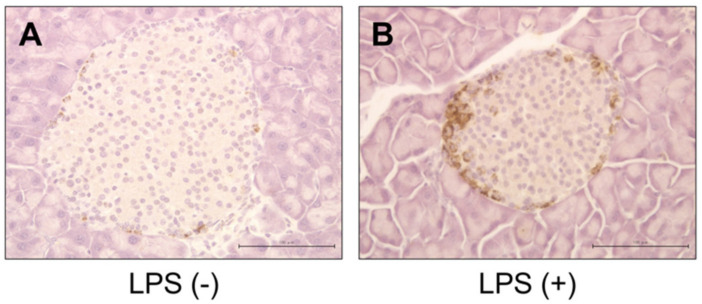
Immunohistological staining for *Reg3A/G* in the *P. gingivalis* lipopolysaccharide (PG-LPS) and control mice. Strongly positive staining for *Reg3A/G* was observed in the islets of Langerhans in the LPS-administered mice. Scale bar, 100 µm. (**A**) PG-LPS(−). (**B**) PG-LPS(+).

**Figure 4:**
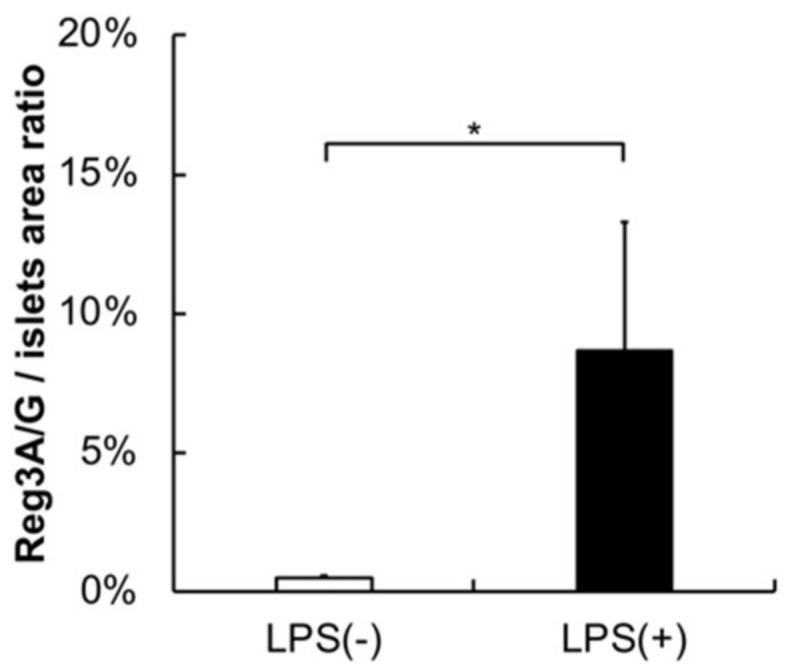
*Reg3A/G*/Islets area ratio from image analysis. Image analysis showed that the ratio of *Reg3A/G*-positive cells was significantly higher in the PG-LPS group than the control. Data are shown as mean ± standard deviation (SD), Mann–Whitney *U* test (* *p* ˂ 0.05; *n* = 5).

**Figure 5:**
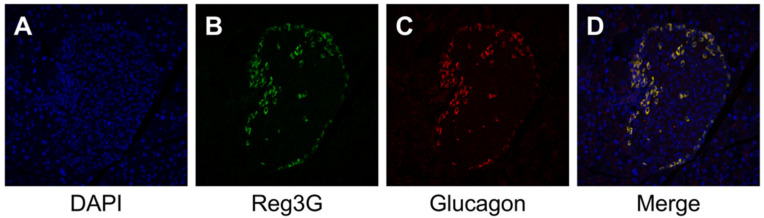
Immunochemical staining for *Reg3A/G* and alpha-cell in the *P. gingivalis* lipopolysaccharide (PG-LPS) mice. Immunofluorescence staining showed the presence of *Reg3A/G*-positive cells in the alpha-cell equivalent areas around the islets of Langerhans in the PG-LPS group. (**A**) 4′,6-diamidino-2-phenylindole (DAPI). (**B**) *Reg3A/G*. (**C**) Glucagon. (**D**) Merge.

No significant inflammatory changes were observed in the control group or PG-LPS group (Figure 2A,B). Strongly positive staining for *Reg3G* was observed in the islets of Langerhans in the LPS-administered mice (Figure 3A,B). Image analysis showed that the ratio of *Reg3A* and *G*-positive cells was significantly higher in the PG-LPS group than the control (Figure 4; *p < 0.05). Immunohistochemical staining showed *Reg3A* and *G*-positive cells in the alpha-cell equivalent areas around the islets of Langerhans in the PG-LPS group (Figure 5A–D).

## 3. Discussion

In the present study, we observed the effect of PG-LPS on the pancreas in an attempt to establish a mouse model that is not affected by acute inflammation in various organs; the gene expression of the pancreas was exhaustively analyzed in this model. Based on the results of the microarray analysis, *Ighg3*, *S100A8*, *S100A9*, *LOC102642252*, *Igk*, *Iglv1*, *Reg3G*, *Igkv4-53*, *Ighv10-3*, and *Chil3/Chil4* were the top 10 genes with the highest expression levels (above 45-fold change) in the pancreas of mice stimulated with PG-LPS (Table 1). *Reg3A* and *G* have been involved in the development and progression of pancreatic cancer [14]. *Reg3A* demonstrated a 11.256-fold change in the microarray data, and the upregulation was confirmed by qRT-PCR in the current study. The upregulated expression levels of *Reg3A* and *G* might play a key role in PG-LPS-related pancreatic cancer in mouse.

The regenerating (Reg) protein family comprises C-type lectin-like proteins that are initially discovered in the pancreas and are expressed in multiple organs [15]. Reg family proteins including *Reg1A, 1B, 3A, 3G*, and *4* act as anti-inflammatory, antiapoptotic, and mitogenic agents in multiple physiological and disease conditions [16]. The *Reg3* proteins have promoted pancreatic islet growth in response to inflammation and injury [17]. In addition, *Reg3* may be involved in the progression of precancerous lesions and cancer in the pancreas [18]. The expression of *Reg3A* was significantly upregulated in pancreatic, liver, and colorectal cancer in humans [19,20,21], while significant upregulation of *Reg3G* has been specifically noted in pancreatic cancer [13]. The *Reg3A* and *G* proteins share 85% sequence homology [22] and are often collectively expressed as *Reg3A/G*. The expression levels of *Reg3A* and *B* may be overlapped. The upregulated expression levels of *Reg3A/G* stimulated with PG-LPS in the present study imply that these proteins may be key for periodontal disease-related pancreatic cancer in human. A recent study showed that *Reg3A/G* were highly expressed in pancreatic acinar–ductal metaplasia, but not in healthy or cancerous pancreas; *Reg3A* protein was found to promote pancreatic duct metaplasia in a 3D culture of mouse acinar cells [17]. *Reg3G* has been reported to function as an immunosuppressive promoter by suppressing the antitumor effects of T cells in a murine model of pancreatic cancer [13]. These findings indicate that *Reg3A/G* may be indirectly involved in carcinogenesis by inducing the development of precancerous lesions or via immunosuppression.

Immunohistochemical staining revealed co-localization of *Reg3A/G*- and glucagon-positive cells around the pancreatic islets in the PG-LPS group. In a previous study, *Reg3A* was found to be co-localized with alpha cells in islets of Langerhans in mice [23]. In addition, mRNA expression levels of *Reg3A* were upregulated by stimulation with inflammatory cytokines, such as *IL-6* [24]; *Reg3G* was not detected in islets but was detected in the total pancreas [24]. Immunohistochemical co-localization with glucagon-positive cells may be mainly affected by *Reg3A* expression. *Reg3G* has been identified as a pancreatitis-associated protein released by the acini during acute pancreatitis and injury [25]. In the current study, no obvious inflammatory infiltration or injury was noted in the pancreas, and no immunohistochemical expression of *Reg3A/G* was observed in the acini. The upregulated expression of *Reg3G* may not be related to inflammatory damage; nonetheless, it might be of importance in the PG-LPS-related pancreatic response.

The mechanism by which PG-LPS induces the upregulation of both *Reg3A* and *G* remains unknown. PG-LPS is recognized by TLR2 and 4 [10], whereas LPS derived from *E. coli* is recognized by TLR2 [9]. TLR2 and 4 are found in a diversity of cells of the immune system. Both receptors have been localized in endocrine and exocrine tissues in the pancreas [26]. Several inflammatory cytokines are activated by TLR2 and 4 via NF-kB and MAPK pathways in the cells [27]. Recently, the *P. gingivalis* membrane, which is supposed to contain LPS, was reported to activate both NF-kB and MAPK [28]. LPS derived from *E. coli* induces the upregulated expression of *Reg3G* via the p38 MAPK pathway [29]. These pathways may be involved in the upregulation of *Reg3A* and *G* by PG-LPS stimulation. Further investigations are needed to clarify this phenomenon.

Although periodontal diseases have been shown as a risk factor of pancreatic cancer, their effect on the pancreas remains unclear. In the present study, we firstly detected a key molecule, the *Reg3A/G*, which might be involved in this phenomenon and could be a target for the prevention of periodontal-related pancreatic cancer. Recently, *Reg3G* was detected in urine obtained from experimentally induced chronic pancreatitis [30]. The early detection of *Reg3A/G* in urine or blood may prove useful for the prevention of cancer in patients with periodontal diseases. Our data are expected to be applied in a clinical setting.

## 4. Materials and Methods

### 4.1. Animals

All procedures involving animals were performed according to the Regulations for the Care and Use of Laboratory Animals at the Health Sciences University of Hokkaido. This experimental protocol was approved by the animal experimental committee and ethics committee of the Health Sciences University of Hokkaido (Permission number: 089, 22/06/2018). PG-LPS (lipopolysaccharide from *P. gingivalis* ATCC 33277; Wako, Osaka, Japan) was prepared in physiological saline and intraperitoneally administered to C57BL/J mice (age 6–8 weeks; Sankyo Labo Service Corporation, Sapporo, Japan) at a dosage of 5 mg/kg every 3 days (84 h) for a period of 1 month (10 doses). Additionally, a control group was administered with the same volume of physiological saline without PG-LPS using the same schedule. Both groups were fed at standard laboratory chow and allowed free access to water in an air-conditioned room (*n = 5* in each group). Anesthesia was carefully induced by intraperitoneal injection of pentobarbital (Somnopentyl^®^, Kyoritsu Seiyaku Co., Tokyo, Japan; 100 mg/kg body weight).

### 4.2. RNA Extraction and Microarray

Three days after the last injection, the mice were sacrificed by cervical dislocation under deep anesthesia, and the pancreases were extracted. Total RNA was extracted from the pancreas using RNeasy^®^ Mini kit (Qiagen GmbH, Hilden, Germany) according to the manufacturer’s instructions, and microarray was performed with SurePrint G3 Rat GE 8x60K Ver.2.0 (Agilent Technologies, Ltd., Santa Clara, CA, USA).

### 4.3. Quantitative Reverse Transcriptase-Polymerase Chain Reaction (RT-PCR)

The genes which showed the highest level of expression in microarray analysis were further confirmed by real-time RT-PCR. Reverse transcription of the extracted RNA was done using ReverTra Ace^®^ qPCR RT Master Mix (Toyobo, Osaka, Japan). Quantitative real-time PCR was performed to demonstrate the expression level of mRNA using LightCycler^®^ Nano (Roche Diagnostics, Basel, Switzerland). The reaction mix for PCR consisted of cDNA, a pair of primer and KAPA SYBR FAST qPCR Mix (Nippon Genetics Co. Lid., Tokyo, Japan). PCR was performed under the following conditions: initial incubation at 50 °C for 2 min, denaturation at 95 °C for 10 min, 40 cycles of denaturation at 95 °C for 15 s, and annulation at 60 °C for 1 min. The relative mRNA expression level was calculated as the Cq obtained after subtracting the Cq value of GAPDH from the Cq value of the target gene using the ∆∆Cq method [31]. The primer sequences used in this study are shown in Table 2.

### 4.4. Histological, Immunohistochemical, Immunofluorescence, and Morphological Analyses

The pancreas obtained from the mouse was fixed in 10% neutral buffered formalin for 24 h. It was then embedded in paraffin and coronal sections of 5 µm thickness were made. The sections were stained with hematoxylin–eosin (H & E) for morphological analysis. Images of the stained sections were taken using OLYMPUS BX50 (Olympus, Tokyo, Japan) using FLOVEL Filing System camera (Flovel, Tokyo, Japan).

For immunohistochemistry, sections were deparaffinized in xylene and rehydrated in ethanol. The peroxidase activity was removed with 3% H_2_O_2_/methanol for 10 min at room temperature (RT) which was then blocked with 3% bovine serum albumin. The sections were incubated for 60 min at 37 °C after the addition of primary anti-Reg3G antibody (ab198216; Abcam, Cambridge, UK; dilution, 1:200). The sections were then washed with PBS and incubated for 30 min at 37 °C after addition of secondary antibody EnVision + System-horseradish peroxidase (HRP)-labeled polymer goat anti-rabbit *IgG* (K4002; Dako North America, Inc., CA, USA) or EnVision + System-HRP-labeled polymer goat anti-mouse IgG (K4000; Dako North America, Inc.). DAB Peroxidase (HRP) Substrate Kit (DAKO Japan, Tokyo, Japan) was used to visualize the staining which produced brown color. Counterstaining with hematoxylin was done to stain the cell nuclei. Images were taken using OLYMPUS BX50 (Olympus, Tokyo, Japan) using a FLOVEL Filing System camera (Flovel, Tokyo, Japan). Binary image thresholding is the most commonly used technique to quantitatively examine changes in immunolabeled material [32]. We used ImageJ software (National Institutes of Health, Bethesda, MD, USA) to quantify the area of *Reg3A* and *G*-positive cells in pancreatic islets by binarizing image date followed by area extraction.

For immunofluorescence, the sections were first blocked with 3% normal goat serum for 30 min. The sections were incubated with primary anti-*Reg3G* antibody (ab198216; Abcam, Cambridge, UK; dilution, 1:100) and primary anti-Glucagon antibody (ab36232; Abcam, Cambridge, UK; dilution, 1:100) for 60 min at RT. Secondary antibody, Goat anti-rabbit *IgG* (H+L) (AF488, Thermo Fisher Scientific, Tokyo, Japan; dilution, 1:100) or Donkey Anti-Sheep *IgG* (H+L) (CF543, Biotium, CA, USA; dilution, 1:100) according to the species of primary antibodies were then added and incubated for 30 min at RT. DAPI (SouthernBiotech, AL, USA) was used to stain the cell nuclei. Sections were visualized with a confocal microscope (C1siReady, NIKON, Tokyo, Japan).

### 4.5. Statistical Analysis

Statistical analysis was performed using SPSS version 23 software (SPSS, Inc., Chicago, IL, USA). The results were compared using Mann–Whitney *U* test. Data are presented as mean ± standard deviation (SD) with *p* < 0.05 considered as significant.

## Figures and Tables

**Table 1:** Upregulated genes in the *P. gingivalis* lipopolysaccharide-administered vs. control animals.

Gene Symbol	Protein Name	Fold Change (Control vs LPS)
*Ighg3*	Immunoglobulin heavy constant gamma 3	4123.958
*S100A8*	S100 calcium-binding protein A8	523.053
*S100A9*	S100 calcium-binding protein A9	336.474
*LOC102642252*	Immunoglobulin heavy chain variable region	263.940
*Igk*	Immunoglobulin kappa chain	241.758
*Iglv1*	Immunoglobulin lambda variable 1	112.254
*Reg3G*	Regenerating islet-derived 3 gamma	73.305
*Igkv4-53*	Immunoglobulin kappa variable 4-53	66.337
*Ighv10-3*	Immunoglobulin heavy variable V10-3	51.897
*Chil3/Chil4*	Chitinase-like 3/Chitinase-like 4	48.847

**Table 2:** Real-time polymerase chain reaction (PCR) primer sequences used in this study.

Gene	Forward	Reverse
*GAPDH*	AGAACATCATCCCTGCATCC	CACATTGGGGGTAGGAACAC
*Reg3A*	TTCCTTTGTGTCCTCCTTGG	ACCTCCATTGGGTTGTTGAC
*Reg3G*	AACAGAGGTGGATGGGAGTG	GTGATTGCCTGAGGAAGAGG

## Abbreviations

*Chil3/Chil4*	*Chitinase-like 3 / Chitinase-like 4*
*E. coli*	*Escherichia coli*
H & E	Hematoxylin–eosin
HRP	Horseradish peroxidase
*Ighg3*	*Immunoglobulin heavy constant gamma 3*
*Ighv10-3*	*Immunoglobulin heavy variable V10-3*
*Igk*	*Immunoglobulin kappa chain*
*Igkv4-53*	*Immunoglobulin kappa variable 4-53*
*Iglv1*	*Immunoglobulin lambda variable 1*
LPS	Lipopolysaccharides
*P. gingivalis*	*Porphyromonas gingivalis*
PG-LPS	*P. gingivalis* lipopolysaccharide
RT-PCR	Quantitative reverse transcriptase-polymerase chain reaction
*Reg*	*Regenerating*
*Reg3G*	*Regenerating islet-derived 3 gamma*
*S100A8*	*S100 calcium binding protein A8*
*S100A9*	*S100 calcium binding protein A9*
SD	Standard deviation
TLR4	Toll-like receptor 4
